# Transcriptomic Response of Chinese Yew (*Taxus chinensis*) to Cold Stress

**DOI:** 10.3389/fpls.2017.00468

**Published:** 2017-04-28

**Authors:** Delong Meng, Xianghua Yu, Liyuan Ma, Jin Hu, Yili Liang, Xueduan Liu, Huaqun Yin, Hongwei Liu, Xiaojia He, Diqiang Li

**Affiliations:** ^1^School of Minerals Processing and Bioengineering, Central South UniversityChangsha, China; ^2^Key Laboratory of Biometallurgy of Ministry of Education, Central South UniversityChangsha, China; ^3^School of Biology and Environmental Science, University College DublinDublin, Ireland; ^4^The Administrative Centre for China's Agenda 21Beijing, China; ^5^Key Laboratory of Forest Ecology and Environment of State Forestry Administration, Institute of Forest Ecology, Environment, and Protection, Chinese Academy of ForestryBeijing, China

**Keywords:** *Taxus chinensis*, transcriptome, DE isoforms, cold stress, ROS, cold response

## Abstract

*Taxus chinensis* is a rare and endangered shrub, highly sensitive to temperature changes and widely known for its potential in cancer treatment. How gene expression of *T. chinensis* responds to low temperature is still unknown. To investigate cold response of the genus *Taxus*, we obtained the transcriptome profiles of *T. chinensis* grown under normal and low temperature (cold stress, 0°C) conditions using Illumina Miseq sequencing. A transcriptome including 83,963 transcripts and 62,654 genes were assembled from 4.16 Gb of reads data. Comparative transcriptomic analysis identified 2,025 differently expressed (DE) isoforms at *p* < 0.05, of which 1,437 were up-regulated by cold stress and 588 were down-regulated. Annotation of DE isoforms indicated that transcription factors (TFs) in the MAPK signaling pathway and TF families of NAC, WRKY, bZIP, MYB, and ERF were transcriptionally activated. This might have been caused by the accumulation of secondary messengers, such as reactive oxygen species (ROS) and Ca^2+^. While accumulation of ROS will have caused damages to cells, our results indicated that to adapt to low temperatures *T. chinensis* employed a series of mechanisms to minimize these damages. The mechanisms included: (i) cold-enhanced expression of ROS deoxidant systems, such as peroxidase and phospholipid hydroperoxide glutathione peroxidase, to remove ROS. This was further confirmed by analyses showing increased activity of POD, SOD, and CAT under cold stress. (ii) Activation of starch and sucrose metabolism, thiamine metabolism, and purine metabolism by cold-stress to produce metabolites which either protect cell organelles or lower the ROS content in cells. These processes are regulated by ROS signaling, as the “feedback” toward ROS accumulation.

## Introduction

*Taxus chinensis* is a woody shrub, which belongs to the Genus *Taxus* and Family *Taxaceae*. Members of the genus *Taxus* are particularly well-known for their important biological compounds such as taxol and paclitaxel—effective drugs used in many cancer therapies (Hao et al., 2008). The species in the *Taxus* genus are rare and endangered species and are highly sensitive to temperature changes including low temperature. The limited resources call for a study into the species' cold transcriptomic response. Cold stress is a very important abiotic stress limiting plant growth, productivity, and distribution. Significant economic losses have resulted from unusual sudden temperature changes in winter and later cold spring events (Yadav, 2010). Cold stress inhibits plant development directly and, indirectly, through osmotic, oxidative, and other stresses (Thomashow, 1999). The oxidative stress caused by cold stress is the product of accumulation of cold stress responsive messengers—reactive species (ROS). The ROS are produced for the purpose of signaling, while they are also recognized as toxic by-products of aerobic metabolism. ROS trigger signal transduction pathways such as mitogen activated protein kinase (MAPK) signaling path for specific responsive gene expressions. Excessive toxic ROS are scavenged by enzymes such as SOD, CAT, and POD (Bailey-Serres and Mittler, 2006).

Plants show a range of responses to cold stress. The responses include the changes in plasma membrane composition and transport activity, detoxification of ROS, and synthesis of cytoplasm-protectant molecules (Beck et al., 2004; Wang et al., 2013), and involve changes in the expression level of cold-responsive genes. The signaling transduction pathways from cold stress to altering the expression of cold responsive genes is crucial for the response of plants to cold stress. A generic signal transduction includes signal perception, generation of second messengers such as ROS and final transcription regulation by transcription factors (TFs) (Singh et al., 2002; Rahaie et al., 2013). The most important signaling pathway for regulation of cold responsive genes is the MAPK signaling pathway which involves several families of TFs (Pitzschke et al., 2009), second messengers (ROS and Ca^2+^) and plant hormones such as abscisic acid and salicylic acid and ethylene. The ROS induces expression of TFs directly or indirectly by accumulation of plant hormones, and TFs and plant hormones feedback on ROS by regulating expression of genes related to ROS scavengers, such as POD and SOD to minimize the potential damages caused by ROS (Tarkowski and Van den Ende, 2015). It is, therefore, important to investigate gene transcriptional changes to understand gene regulation mechanisms in response to cold stress.

Transcriptome sequencing or RNA-seq, was developed as a powerful tool to obtain an overall view of gene expression profiles of organisms (Ozsolak et al., 2009; Nagalakshmi et al., 2010). The transcriptome of the genus *Taxus* has been previously reported for gene expression profiles in leaf, stem, and root tissues using Illumina sequencing (Hao et al., 2011) and in needles using 454 pyrosequencing (Wu et al., 2011). However, information on the large-scale regulation of gene expression profiles in response to cold stress is still lacking for this temperature-sensitive genus. In the present study, we applied Illumina Miseq sequencing to study the whole transcriptome of Chinese yew (*T. chinensis*) under cold stressed conditions, with a particular focus on the role of ROS-related processes in the stress response. The activity of three ROS scavengers was also analyzed.

## Materials and methods

### Plant material and enzyme activity

Two to three-year old Chinese yew (*T. chinensis*) seedlings were grown in a green house with the settings at 12 light/12 h dark, and a temperature of 23/15°C. The relative humidity was 70% and photosynthetically active radiation at the leaf level was 250–350 μmol m^−2^ s^−1^. Before stress, stems of the control-grown seedlings were wrapped by cotton and cling film and the seedlings were transferred into the growth chamber. The stress treatment was carried out in a growth chamber, with a three-step incubation of plants at 10°C for the first 48 h, followed by 5°C for the next 48 h, and then 0°C until the samples harvest. After plants had been stressed for 12 h at 0°C, plant tissues were harvested for RNA extraction. Tissues from control seedlings and stressed seedlings were sampled from the longest leaf (leaf 5–8). Each treatment (control and cold stress) was carried out three times, resulting in three biological replicates. Collected samples were frozen in liquid nitrogen immediately and stored at −80°C for further molecular analysis.

Activity of three enzymes, superoxide dismutase (SOD), peroxidase (POD), and catalase (CAT) was determined in leaf tissue samples on the Epoch Microplate Reader (BioTek, USA) using an ELISA enzyme activity Kit (TianGen Inc., Beijing, China), following the manufacturers' instructions. The leaf tissue samples were harvested and enzyme activities were measured after 1, 2, 4, 8, 12, 24, and 48 h of the 0°C treatment. Enzyme activities of the control plants (control) were also measured by directly sampling leaf tissues from seedlings that were grown at normal temperature (23°C). Three biological replicates were analyzed.

### RNA extraction, cDNA synthesis, and sequencing

Frozen tissue samples were ground in liquid nitrogen into fine powder, which was used for RNA extraction. Total RNA was extracted using the Column Plant RNAout Kit (Tiandz, Inc. Beijing, China), and genomic DNA was digested using Desoxyribonuclease I Amplification Grade (Invitrogen, San Diego, California, USA) following the manufacturer's instructions. RNA concentration and purity was determined Nanodrop Spectrophotometer (ND-1000 Spectrophotometer, Nanodrop products, Wilmington, USA), and Qubit 2.0 (Thermo Fisher Scientific, USA). Poly(A) mRNA was enriched using magnetic beads with oligo(dT). The mRNA library was constructed using the Illumina TruSeq RNA sample prep kit (Illumina, San Diego, CA) following the manufacturers' instructions. Paired-end sequencing was performed using Miseq 150 bp kit (Illumina, San Diego, CA) on Illumina Miseq platform (Illumina, San Diego, CA). Paired-end sequencing was performed using the following Illumina adapters: adapter-R1 5′-AGATCGGAAGAGCACACGTCTGAACTCCAGTCA-3′ and adapter-R2 5′-AGATCGGAAGAGCGTCGTGTAGGGAAAGAGTGT-3′. The raw data of Miseq paired-end sequencing was the fastq format.

### Data processing and analysis

The barcodes and adapters in reads were removed before assembly, using *cutadapt* (Martin, 2011), and sequencing quality was checked using *Fastqc*. A total of 4.16 Gb clean data was obtained for all sample reads. As none genome data was available for *Taxus*, a *de novo* RNA-seq assembly was performed toward transcriptome analysis, using *Trinity* platform (Grabherr et al., 2011; Haas et al., 2013) following Trinity users' guild. The transcriptome was assembled from all six samples (three control samples and three cold stressed samples). The quality of the transcriptome was assessed by TrinityStats scripts and blasting the transcriptome to Swissprot database (Gasteiger et al., 2001). Before building a transcript expression matrix, transcript abundance in each sample was estimated by aligning each sample reads to the transcriptome using Trinity scripts and combined RSEM (Wang et al., 2009) and bowtie packages. To identify transcripts expressed differently, the transcript expression abundance was normalized by TMM (Trimmed Mean of M-values) method (Robinson and Oshlack, 2010; Dillies et al., 2012). The transcript matrix and normalized TMM matrix were used for downstream analysis such as quality check of samples and replicates and different expression (DE) analysis.

To assess the quality of replicates across all samples, the trinity PtR script was used to generate a correlation matrix (heat map) for all sample replicates using the counts-per-million (CPM) and log2 transformed data. Principal Component Analysis was also performed to explore any relationship among the sample replicates. For DE analysis, the Bioconductor R package, edgeR, was used to perform pairwise comparison between control and cold-stressed samples (Robinson et al., 2010). MA and volcano plots were generated to show transcripts that were expressed differently. Subsequently, all isoforms that were at least 2^∧^2 fold differently expressed and had *p* values at most 0.001, 0.01, and 0.05 respectively, were extracted. Heat maps were made to (i) describe the sample correlations using DE isoforms and (ii) show all isoform correlations in each sample.

Open Reading Frames (ORFs) of transcriptome and DE transcripts (both up- and down-regulated isoforms) at *p* = 0.05 were predicted by Transdecoder (https://github.com/TransDecoder/TransDecoder/releases). ORFs were used for function annotations. KEEG pathway annotation was performed on the KAAS (KEEG Automatic Annotation Serve) website (http://www.genome.jp/tools/kaas/). Gene Ontology (GO) annotation analyses of differently expressed isoforms was performed using BinGo (https://www.psb.ugent.be/cbd/papers/BiNGO/Home.html) by assigning the annotation to the *Arabidopsis*. Plant transcription factors (TFs) were predicted using PlantTFDB database (http://planttfdb.cbi.pku.edu.cn/).

The reads data has been submitted to NCBI SRA database. The accession numbers of reads data in were SRR5167062, SRR5167061, SRR5167060, SRR5167059, SRR5167058, and SRR5167057 in Bioproject PRJNA360896. Other statistical analyses were carried out using the Minitab 16.0 software (Minitab Inc.). A *p*-value of smaller than 0.05 was considered as significant.

### RT-qPCR

RT-qPCR was carried out to validate the RNA-seq data. Digestion of genomic DNA and synthesis of cDNA were similar as in section RNA extraction, cDNA synthesis, and sequencing. Quantitative PCR experiments were performed on Bio-Rad iQ5 Optical System (BIO-RAD Laboratories Inc., USA) using SYBR Premix, and relative expression was calculated as described previously (Meng et al., 2016; Meng and Fricke, 2017). Primers used in the present study were designed using PrimeQuest Tools, and primer sequences are given in Table S1. A linear correlation analysis of qPCR and RNA-seq (Figure S1) showed that expression data obtained from different approaches correlated well with each other (Pearson's correlation = 0.712, and *p* < 0.001).

## Results

### Enzyme activity

**Figure 1:**
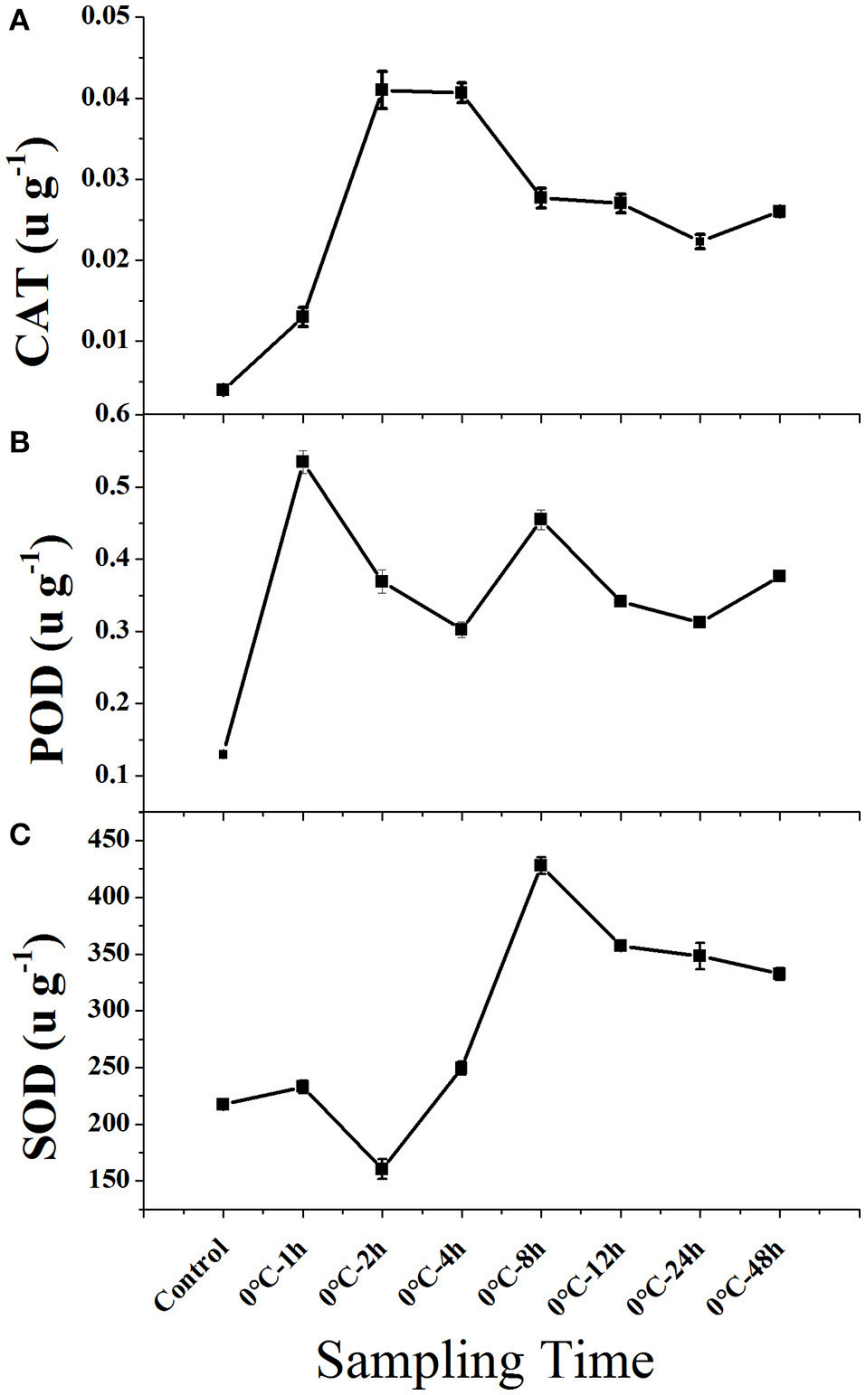
**Enzyme activity of CAT, POD, and CAT in leaf tissues of *Taxus chinensis* grown under normal and cold stress conditions**. **(A)** Hydroperoxidase (CAT), **(B)** Peroxidase (POD), and **(C)** Superoxide Dismutase (SOD). Leaf tissues were sampled from plants grown under normal conditions and from plants that were subjected to 0°C for 1, 2, 4, 8, 12, 24, and 48 h. Results are means and *SD* for three replicates.

Cold stress caused a general increase in the activity of all three enzymes in leaf tissue (Figure 1). Hydroperoxidase (CAT) and peroxidase (POD) activity increased rapidly within 1–2 h when plants were exposed to 0°C and remained at a relative high level. Compare to CAT and POD, superoxide dismutase (SOD) activity increased initially slower, but increased rapidly after 2 h and remained at a high level.

### Transcriptome

The *de novo* assembled transcriptome had a GC content of 41.83% and included 83,963 transcripts of which 62,654 genes were identified. The median and N50 length of the transcriptome was 598 and 1,497 bp, respectively. Of all transcripts assembled, 55,135 transcripts could be annotated in reviewed Uniprot database at expectation value (e) of 1e-5, which account for 65.7% of the total transcripts. A total of 44,368 ORFs were predicted for the transcriptome by Transdecoder, of which 39,672 sequences (89.4%) could be annotated in reviewed Uniprot database at an expectation value of 1e-5.

Principal components analysis (PCA, Figure S2) of CPM showed that (i) replicates of stressed samples were well-grouped, (ii) while replicates of control samples were not as well-grouped, yet they clearly grouped different from stressed samples. Similar results were obtained by correlation matrix heat map analysis.

### Different expression (DE) analyses

**Figure 2:**
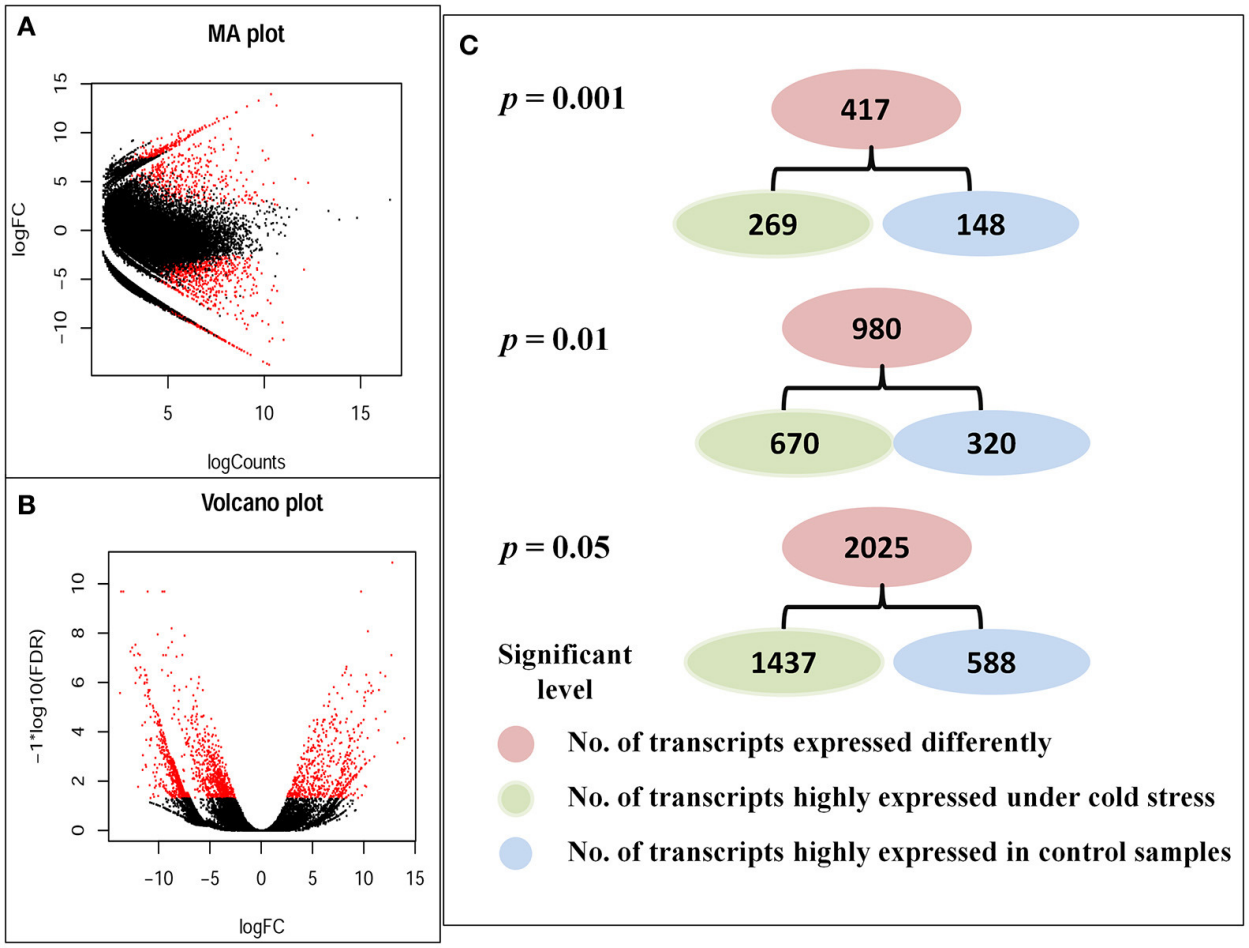
**Differently expressed transcripts between control and cold stressed samples**. **(A)** MA plot, **(B)** Volcano plot, and **(C)** Number of transcripts that expressed most differently at different significant levels. The red dots in MA and volcano plots indicated the transcripts that were differently expressed at least a fold change ratio of four (|log_2_FC| > 2) between control and cold stressed samples.

Different expression (DE) analyses revealed that 37,934 transcripts expressed differently between control and cold stressed samples at a fold-change (FC) ratio of more than 4 (|log_2_FC| > 2), as indicated by red dots in Volcano and MA plots (Figures 2A,B). Of all transcripts that expressed differently, 29,783 transcripts (~78.5%) were up-regulated and 8,151 (~21.5%) were down-regulated under cold stress. A fold-change of more than four (|log_2_FC| > 2) was shown for 417 DE transcripts at *p* < 0.001, 980 transcripts at *p* < 0.01 and 2,025 transcripts at *p* < 0.05 (Figure 2C).

Analysis of sample correlation (Figure S3A) using differently expressed isoforms revealed a close correlation between replicates in each treatment, whereas the relationship between treatments were distant from each other. Isoform features correlation heatmap (Figure S3B) showed that DE isoforms could be partitioned into clusters with similar expression patterns.

### Functional annotation

Annotations were made for 1,437 up-regulated and 588 down-regulated transcripts under cold stress, which had a |log_2_FC| > 2 and a significant level of *p* value < 0.05. Following the prediction by Transdecoder, 1,424 ORFs were predicted from 1,437 up-regulated transcripts and 521 were predicted from 588 down-regulated transcripts.

#### Transcription factors (TFs) prediction

**Figure 3:**
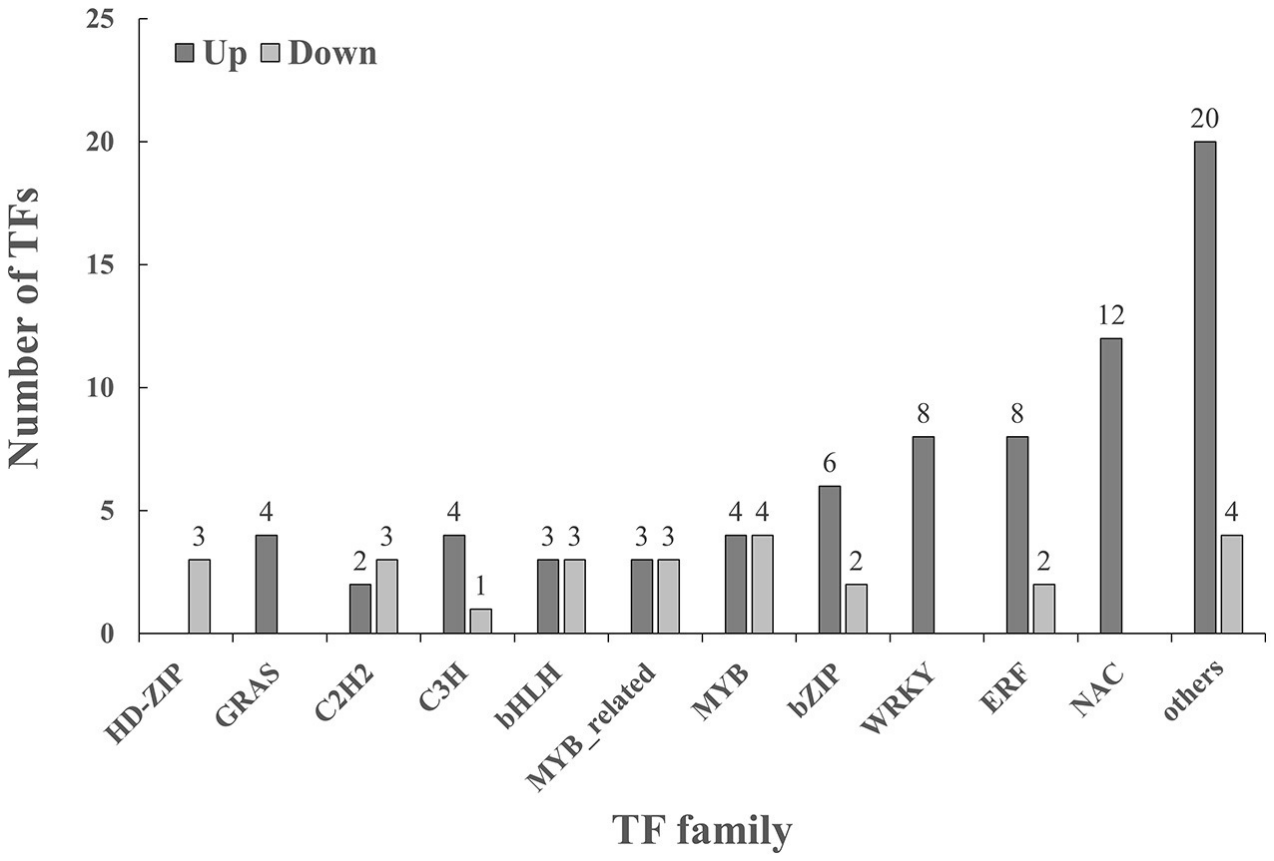
**Classification of transcription factors (TFs) that were significant (*p* < 0.05 and |log2FC| > 2) changed under cold stressed conditions**. Others in cold up regulated include: AP2(1), ARR-B(1) B3(1), BES1(1), Dof(1), E2F/DP(1), G2-like(1) GATA HSF(1), LBD(1), M-type MADS(1), NF-YA(1), NF-YC(1), Nin-like(1), Trihelix(1), CO-like(1), ARF(2), and TALE(2); Others in cold: DBB(1), NF-YB(1), SBP(1), and CO-like(1). Up, cold up regulated; Down, cold down regulated.

Under cold stressed conditions, 74 TFs were up-regulated in leaf tissues of *T. chinensis* and 25 TFs were down-regulated as shown in Figure 3. Cold up-regulated TFs belonged to 28 TF families. The NAC, ERF, bZIP, and MYB family contained most up-regulated TFs and had 12, 8, 8, 6, and 4 TFs, respectively. Cold down-regulated TFs belonged to 12 different families, and the five most abundant families were MYB, bHLH, C2H, MYB-related, and the HD-ZIP family.

#### KEGG pathway classification

**Figure 4:**
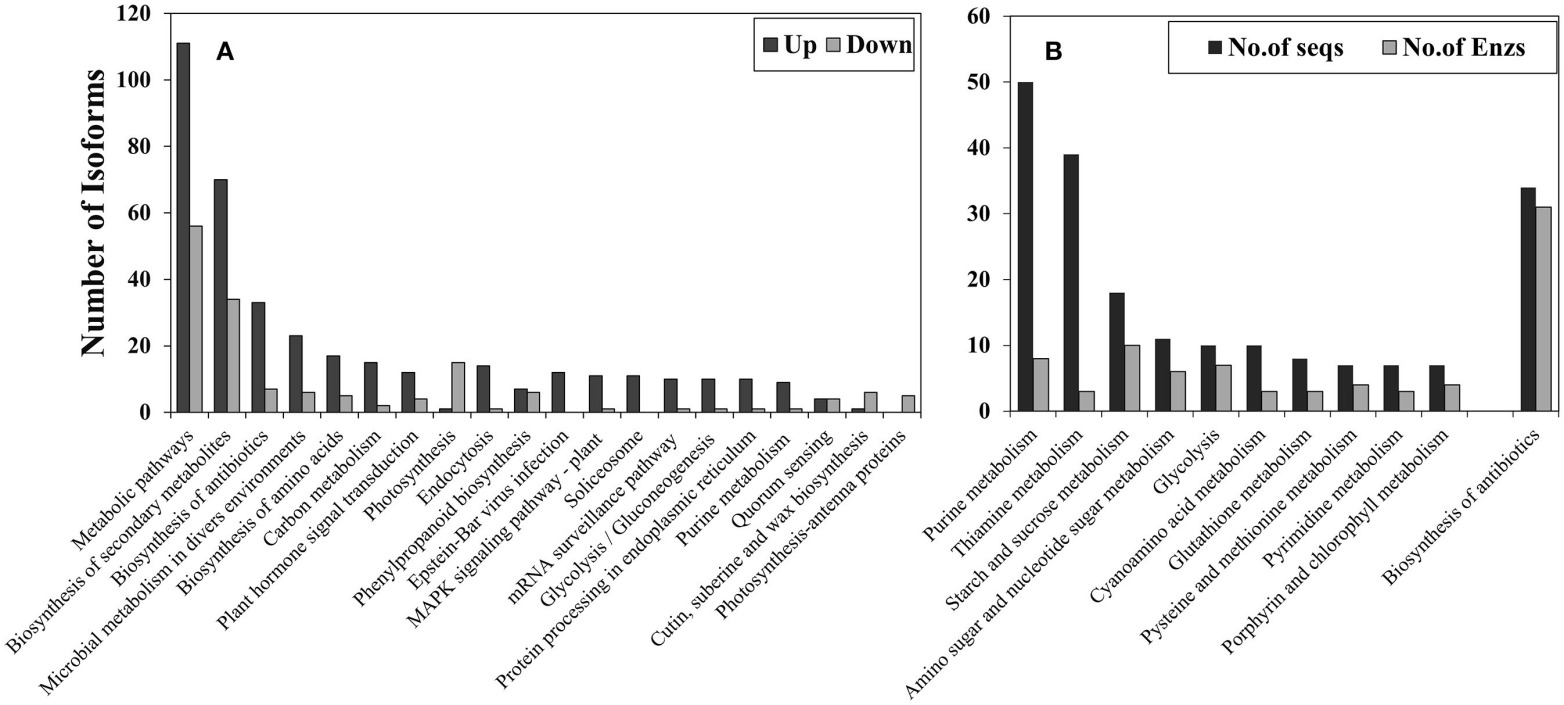
**KEGG path way annotation for transcripts that were significantly (*p* < 0.05) affected by cold stress and had at least a fold-change of four (|log_2_FC|> 2)**. **(A)** Overall classification and **(B)** sub-classification of metabolic pathways. Up, cold up-regulated transcripts; Down, cold down-regulated transcripts.

KEGG pathway classification (Figure 4A) showed that most significantly-changed transcripts belonged to metabolic pathways (111 up-regulated and 56 down-regulated) and the biosynthesis of secondary metabolites (70 down-regulated and 34 down-regulated). Most of the pathways had more up-regulated transcripts than down-regulated transcripts. The only pathway which had more down-regulated transcripts was the photosynthetic pathway. In addition, KEGG annotation showed that the plant mitogen-activated protein kinases (MAPK) signaling pathway (Ko04016) was significantly affected by cold stress, and most of the changed gene isoforms in the MAPK signaling pathway were up-regulated whereas only one isoform was down-regulated. Eleven genes were up-regulated in the MAPK signaling pathway (Figure S4), and these genes were MEKK1(K13414), MEKK9 (K20604), WRKY33 (K13424), NDPK2 (K00940), ETR/ERS (K14509), ChiB (K20547), YC2 (K13422), PYR/PYL (K14496), PP2C (K14497), CaM4 (K02183), and YODA (K20717). Most of these genes locate up-stream of MAPK signaling pathway.

Sub-classification of cold-up regulated metabolic pathways (Figure 4B) showed that 50 transcripts belonged to purine metabolism, with eight enzymes being identified, 39 transcripts belonged to thiamine metabolism (three enzymes), 18 transcripts belonged to starch and sucrose metabolism (10 enzymes), and 11 transcripts in amino and nucleotide sugar metabolism (eight enzymes).

#### GO annotation and classification

**Figure 5:**
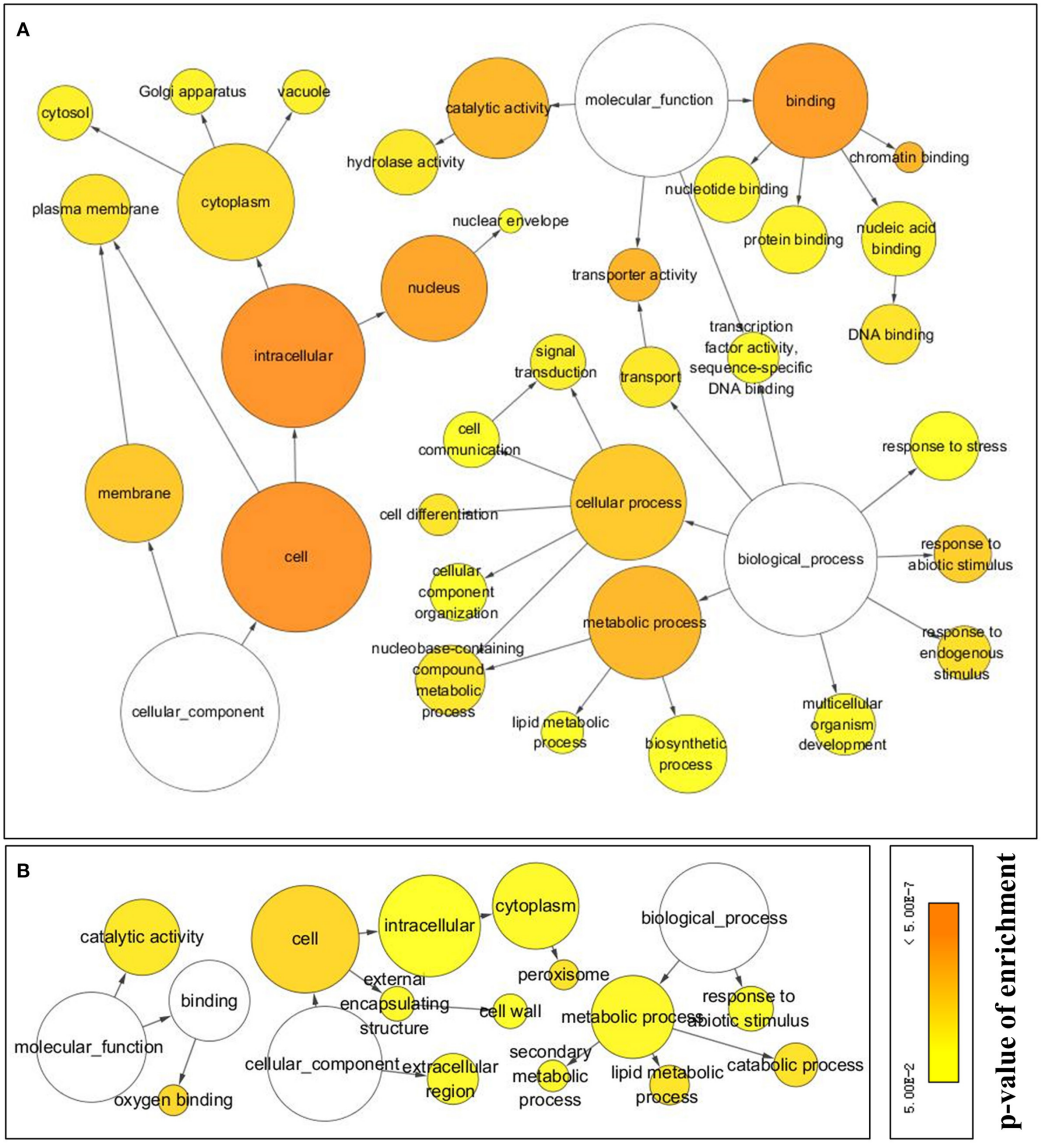
**Networks of GO terms enriched in DE ORFs at *p* < 0.05 level and had at least a 4-folderexpression ratio change (log_2_FC > 2) in response to cold stress**. **(A)** GO terms enriched from cold up regulated ORFs, **(B)** GO terms enrichd from cold down regulated ORFs. GO enrichment analysis were carried out using BinGo after ORFs annotated to Arabidopsis. The lines between GO terms indicated their parent-child relationships. Sizes of circles represent the size of GO terms in Arabidopsis annotation. Colors indicate *p*-values of Benjamini and Hochberg False Discovery Rate (FDR) correction. Detailed information of GO terms are list in Table 1.

Of the 1,424 up- and 456 down-regulated ORFs, 961 and 341 were assigned to GO terms, respectively, after an annotation based on Arabidopsis homologs. Networks (Figure 5) of GO terms enriched among the DE ORFs, were constructed based on their parent-child relationships and network features were shown in Table 1.

**Table 1:** **Summary of gene ontology (GO) enrichment of differently expressed isoforms in *Taxus chinensis* in response to cold stress**.

**Cold response**	**GO-ID**	**GO term^a^**	***p*-value^b^**	**Number(%) in DE isoforms^c^**	**Number(%) in background^d^**
Up		(1)MF			
	3824	(1.1)catalytic activity	2.37E-05	348(36.21)	8,943(29.37)
	16787	(1.1.1)hydrolase activity	4.27E-03	130(13.53)	3,152(10.35)
	5488	(1.2)binding	1.62E-06	459(47.76)	11,991(39.38)
	3682	(1.2.1)chromatin binding	1.87E-05	18(1.87)	149(0.49)
	3676	(1.2.2)nucleic acid binding	1.21E-02	174(18.11)	4,533(14.89)
	3677	(1.2.2.1)DNA binding	2.90E-03	105(10.93)	2,412(7.92)
	5515	(1.2.3)protein binding	1.55E-02	141(14.67)	3,613(11.87)
	166	(1.2.4)nucleotide binding	1.53E-02	133(13.84)	3,373(11.08)
	5215	(1.3)transporter activity	1.65E-05	78(8.12)	1,398(4.59)
	6810	(1.3.1)transport	4.27E-03	108(11.24)	2,536(8.33)
	3700	(1.4)transcription factor activity, sequence-specific DNA binding	3.25E-02	72(7.49)	1,727(5.67)
		(2)BP			
	6950	(2.1)response to stress	1.77E-03	145(15.09)	3,473(11.41)
	9628	(2.2)response to abiotic stimulus	2.60E-04	95(9.89)	1,978(6.50)
	9719	(2.3)response to endogenous stimulus	1.77E-03	81(8.43)	1,727(5.67)
	7275	(2.4)multicellular organism development	2.57E-02	112(11.65)	2,833(9.31)
	8152	(2.5)metabolic process	2.35E-05	447(46.51)	11,939(39.2)
	9058	(2.5.1)biosynthetic process	4.99E-02	197(20.50)	5,437(17.86)
	6629	(2.5.2)lipid metabolic process	4.99E-02	44(4.58)	1,002(3.29)
	6139	(2.5.3)nucleobase-containing compound metabolic process	3.94E-03	150(15.61)	3,702(12.16)
	9987	(2.6)cellular process	1.51E-04	470(48.91)	12,880(42.30)
	7165	(2.6.1)signal transduction	9.82E-03	86(8.95)	1,998(6.56)
	7154	(2.6.1.1)cell communication	4.56E-02	89(9.26)	2,242(7.36)
	30154	(2.6.2)cell differentiation	2.90E-03	42(4.37)	774(2.54)
	16043	(2.6.3)cellular component organization	4.56E-02	95(9.89)	2,416(7.94)
	6139	(2.6.4)nucleobase-containing compound metabolic process	3.94E-03	150(15.61)	3,702(12.16)
		(3)CC			
	5623	(3.1)cell	1.31E-14	819(85.22)	22,668(74.45)
	5622	(3.1.1)intracellular	8.30E-10	746(77.63)	20,724(68.07)
	5634	(3.1.1.1)nucleus	3.40E-06	388(40.37)	9,915(32.57)
	5635	(3.1.1.1.1)nuclear envelope	4.56E-02	9(0.94)	121(0.40)
	5737	(3.1.1.2)cytoplasm	1.00E-03	479(49.84)	13,400(44.01)
	5773	(3.1.1.2.1)vacuole	1.11E-02	49(5.10)	1,021(3.35)
	5794	(3.1.1.2.2)Golgi apparatus	1.55E-02	54(5.62)	1,181(3.88)
	5829	(3.1.1.2.3)cytosol	1.15E-02	85(8.84)	1,989(6.53)
	16020	(3.2)membrane	1.18E-04	328(34.13)	850427.93
	5886	(3.2.1)plasma membrane	2.27E-03	153(15.92)	3,724(12.23)
Down		(1)MF			
	3824	(1.1)catalytic activity	4.47E-03	130(24.95)	8,942(29.37)
		(1.2)binding			
	19825	(1.2.1)oxygen binding	6.64E-04	12(2.30)	2,34(077)
		(2)BP			
	9628	(2.1)response to abiotic stimulus	3.56E-02	35(6.72)	1,978(6.50)
	8152	(2.2)metabolic process	2.72E-02	159(30.52)	11,938(39.21)
	9056	(2.2.1)catabolic process	2.39E-03	33(6.33)	1,433(4.71)
	6629	(2.2.2)lipid metabolic process	3.27E-03	25(4.80)	1,002(3.29)
	19748	(2.2.3)secondary metabolic process	2.64E-02	13(2.50)	469(1.54)
		(3)CC			
	5576	(3.1)extracellular region	2.83E-02	49(9.40)	2,954(9.70)
	5623	(3.2)cell	6.64E-04	287(55.09)	22,667(74.45)
	30312	(3.2.1)external encapsulating structure	2.64E-02	17(3.26)	700(2.30)
	5618	(3.2.1.1)cell wall	2.64E-02	17(3.26)	700(2.30)
	5622	(3.2.2)intracellular	4.97E-02	253(48.56)	20,723(68.06)
	5737	(3.2.2.1)cytoplasm	2.83E-02	175(33.59)	13,399(44.01)
	5777	(3.2.2.1.1)peroxisome	2.67E-03	11(2.11)	247(0.81)

a *All GO terms in the networks were presented. Numbers in the brackets indicated the parent-child relationship between the terms, e.g., “(1.1.1)hydrolase activity” is a child term of “(1.1)catalytic activity.” MF, molecular function; BP, biological process; and CC, cellular component*.

b *Significance level of Benjamini and Hochberg False Discovery Rate (FDR) correction*.

c *Percentage of the total classified up/down regulated isoforms (n = 961 and 341 for up and down regulated isoforms, respectively)*.

d *GO annotation of Arabidopsis thaliana (n = 30448)*.

In the category “biological process,” there were three terms, “response to stress” (GO 6950, 15.09%), “response to abiotic stimulus” (GO 9628, 9.89%), and “response to endogenous stimulus” (GO 9719, 8.43%) represented in cold up-regulated ORFs, while the term “response to abiotic stimulus” (6.33%) was also represented in cold down-regulated ORFs. Protein names of cold up-regulated ORFs in the three terms are listed in Table S2.

The most abundant term enriched in cold response ORFs was the “cell” (GO 5623, 85.22 and 55.09% in up and down regulated ORFs, respectively) in the category cellular component. Its child terms “plasma membrane” (GO 5886, 15.92%), “cytoplasm” (GO 5737, 49.84%), and “nucleus” (GO 5634, 49.84%) were enriched in cold up regulated ORFs. In addition, nine up-regulated ORFs were classified into the term “nuclear envelope” (GO 5635)—a child term of “nucleus.”

In terms of the molecular function, two major terms and their child terms were enriched in cold up regulated ORFs, those were “catalytic activity” (GO 3824, 36.21%) and its child term—“hydrolase activity” (GO 16787) and “binding” (GO 5488, 47.76%) and its child terms—chromatin binding (GO 3682, 1.87%), nuclei acid binding (GO 3676, 18.11%), DNA binding (GO 3677, 10.93%), protein binding (GO 5515, 14.67%), and nucleotide binding (GO 166, 13.84%). Whereas, cold down-regulated two major molecular functions: “catalytic activity” (GO 3824) and “oxygen binding” (GO 19825) of parent “binding” term, which had 130 (24.95%), and 12 (2.30%) ORFs, respectively.

## Discussion

### Plasma membrane, the first cold signal acceptor

The plasma membrane is the first major transport barrier of a cell which experiences environmental changes, and a sensitive cold signal receptor system at the plasma membrane would help plants to adjust to stressed conditions. GO enrichment analysis indicated significant changes in the expression of membrane related genes. Genes such as plasma membrane-associated cation-binding protein and plasma membrane proton pump were identified in the up regulated ORFs which were assigned to “response to stress” term (Table S2), indicating significant changes in plasma membrane function in response to cold stress. This caused changes in the following aspects: (a) membrane fluidity/rigidity, (b) membrane permeability of signal receptors e.g., Ca^2+^, and (c) membrane intrinsic protein family (Alonso et al., 1997). In the present study, the *CaM4* gene of *T. chinensis*, was up-regulated in response to cold stress, pointing to an increased intracellular Ca^2+^ concentration. It suggested that membrane permeability of Ca^2+^ receptors was enhanced in response to cold stress to allow more Ca^2+^ entry into the plasma. Ca^2+^ influx was a very important change (initial event) in membrane during cold and (Chinnusamy et al., 2007) suggested the increase of Ca^2+^ influx ability may be induced by accumulation of ROS caused by cold stimulation. The intracellular Ca^2+^ sensors such as calmodulin (CaM) or calcium-dependent protein kinases could bind the Ca^2+^ and then interact with target proteins to activate a series of regulation evens (e.g., MAPK signaling path) for regulating cold-responsive gene expression (DeFalco et al., 2010). Nevertheless, genes such as Calmodulin-like protein and Calcium-dependent protein kinase were up-regulated in response to cold stress (Table S2, ORFs were annotated as AT5G66210, AT1G24620, AT2G38910, and else).

### Signal transduction and transcription factor (TFs) regulation

Although the secondary messengers of ROS signaling are unknown at present, it has been suggested that ROS (i) inhibits phosphatases directly, change the membrane structure to enhance Ca^2+^ influx and also act as the receptors of Ca^2+^, (3) the unknown messengers were accepted by redox-sensitive transcription factors, e.g., MAPK signaling pathway (Miller et al., 2008). What's more, (Moller and Sweetlove, 2010) proposed oxidized peptides as the possible secondary ROS messengers that are also involved in MAPK signaling. It is commonly accepted that the plant MAPK signaling pathway plays an important role in plant abiotic stress responses (Pitzschke et al., 2009; Huang et al., 2012; Danquah et al., 2014). The secondary ROS messenger of the MAPK cascade caused indirect activation/up-regulation of cold-responsive TFs to enhance the tolerance capability to cold. One of the most effective defense response to stress is plant signal regulation by TFs, which are components of complex regulatory network in plants.

Eleven TFs in plant-MAPK signaling pathway were up-regulated by cold stresses as predicted by KEGG pathway annotation, particularly TFs that located upstream of the pathway. It has been reported that plants with over-expressed *AtNDPK2* had reduced ROS levels (Moon et al., 2003), indicating the role of *NDPK2* in regulating ROS scavenging-related genes. This is consistent with the present finding that the increased ROS scavenger activity in *T. chinensis* could be well-explained by the up-regulated *NDPK2* in the MAPK signaling pathway. Two of the TFs in the MAPK pathway, *ETR/ERS* and *CaM4* are secondary signal receptors of ethylene and Ca^2+^, respectively, both substances being messengers induced by stresses. It has been reported for Arabidopsis that the MAPKKK (MAP kinase kinase kinases)-MAPKK (MAP kinase kinases)-MAPK cascade could be activated by ROS, and assist plants in cold acclimation (Teige et al., 2004). Cold stress might have also activated this cascade in *T. chinensis* to regulate gene expression, as the MEKK1 and MKK9 members of the cascade showed significant up-regulation by cold stress. *MYC2* has been reported to be an osmotic responsive gene and to be involved in ABA signaling (Abe et al., 2003) and enhance osmotic stress tolerance of transgenic plants (Gao et al., 2011). The up-regulated *MYC2* in *T. chinensis* might have been due to the osmotic stress component of cold stress and enhanced ABA signaling caused by low temperature. Some studies have supported the idea that PP2C is an important member of the ABA signal transduction pathway and that *PP2C* functions as regulator of various signal transduction pathways (Rodriguez, 1998). Our observation that PP2C is up-regulated by cold stress supports the ideas that PP2C is activated by accumulated ABA.

Many of the downstream TFs in the MAPK signaling pathway were not up-regulated by cold stress by a factor of four. This may be because many other TFs fulfill “complementary” functions of those TFs. Nevertheless, many cold responsive TF families were found to be up-regulated by cold stress by a factor of more than four. Five TF families including AP2-EREBP (APETALA2/ET-Responsive Element Binding Protein), MYB (Myeloblastosis), NAC (NAM, ATAF1/2, CUC2), bHLH (basic Helix-Loop-Helix), and WRKY (named after the WRKY amino acid motif) have been shown to be involved in the cold stress response in *Arabidopsis* (Changsong and Diqiu, 2010). All these five families were affected by cold stress in *T. chinensis*, and therefore, they support a role in the *Taxus* cold stress response. This applies in particular to NAC, WRKY, and MYB which were the three TF families most-affected in expression by low temperature. Two of the other most numerous TF families up-regulated by cold stress in *Taxus* were ERF and bZIP. They have also been reported to be involved in the cold stress response in other species (Heidarvand and Maali Amiri, 2010; Mizoi et al., 2012; Calzadilla et al., 2016).

### Changes in ROS scavenging system

Almost twice as many genes (62,654 vs. 36,493) were assembled in the present study as previously reported (Hao et al., 2011). This may be due to the existence of cold activated genes, as overall, 29,783 transcripts up-regulated under cold stressed condition by a factor of 4 (|log_2_FC|> 2). At all significant levels, more isoforms were up-regulated than down-regulated in response to cold stresses, similar to the findings in wheat (Winfield et al., 2010), *Lotus japonicas* (Calzadilla et al., 2016), and *Arabidopisis* (Fowler, 2002).

Reactive oxygen species (ROS), for example superoxide (${\text{O}}_{2}^{-}$) and H_2_O_2_, play important roles in regulating plant gene expression, particularly under abiotic stress conditions (Chinnusamy et al., 2007; Heidarvand and Maali Amiri, 2010; Tamás et al., 2010; Aroca et al., 2012; Li et al., 2014). It has also been reported that ROS which accumulate under cold stress activate the membrane Ca^2+^ channels to increase Ca^2+^ flux for gene regulation signaling. The roles which ROS play are distinct (del Río et al., 2006; Huang et al., 2012). On the one hand, they are thought to play a key role in regulating stress-induced signal transduction events. On the other aspect, ROS caused many harmful effects as they inhibit proteins such as aquaporins (Knipfer et al., 2011), induce damages to DNA and lipids, and can ultimately lead to cell death. Therefore, a common problem that plants grown under stressed condition face is how to remove or detoxify ROS effectively. Genes that were assigned in the present study to GO terms “Response to abiotic stimulus,” “Response to stress,” and “Response to endogenous” are listed in Table S2. Genes of ROS scavenging enzymes, such as peroxidase (AT5G05340 and else) and phospholipid hydroperoxide glutathione peroxidase (PHGPx, AT4G11600) were transcriptionally up-regulated in response to cold stress. It has been reported that the up-regulation of these genes inhibits cell death induced by ROS (Chen et al., 2004). Also, enzyme activity of ROS scavengers (including SOD, CAT, and POD) increased under cold stress in the present study. It has been suggested that the scavenging system responds positive to cold stress by removing the toxic ROS that are, initially, necessary for inducing cold-responsive gene expression (Bailey-Serres and Mittler, 2006). In the present study, the increase in CAT activity was the highest of the three ROS-related enzymes studied, might due to that H_2_O_2_ as a signaling factor might be the most abundant ROS and other ROS should firstly converse to H_2_O_2_ for further deoxidation. Taken together, the present data suggest that that up-regulation of the genes encoding ROS scavengers and activation of enzymes enhanced the ROS detoxification ability of *T. chinensis* in leaf tissues.

“Metabolism” was the most abundant category in the KEGG classification and the category most affected by cold stress. The most numerous four pathways in this classification were purine metabolism, thiamine metabolism, starch and sucrose metabolism, and amino and nucleotide sugar metabolism.

Purine metabolism is considered as a housekeeping function, however, a recent study by Watanabe et al. (2014) and Takagi et al. (2016) indicated that the purine metabolite, allantoin is involved in stress response such as ABA metabolism and jasmonic signaling, and therefore enhances plants cold tolerance. Our results showed that purine metabolism in *Taxus* was up-regulated. An increase in purine nucleotides in response to cold has been reported previously for plants and animals (Desautels and Himmshagen, 1979; Frischknecht and Baumann, 1985).

Up regulation of thiamine metabolism and starch and sucrose metabolism and amino and nucleotide sugar metabolism in response to cold stress may have played a role in ROS scavenging. It has been reported that thiamine (vitamin B1) is induced by the oxidative stress which accompanies cold stress rather than cold stress *per se* (Rapalakozik, 2011) and that thiamin inhibits lipid peroxidation and free radical oxidation of oleic acid to protect cells from ROS. Some of the carbohydrates are important components of the ROS scavenging system, function together with other components such as phenylpropanoids (Van den Ende and Valluru, 2009). Therefore, changes in starch and sucrose metabolism in response to cold stress may contribute to scavenging of excessive ROS.

## Conclusion

In conclusion, the present study is the first insight into the transcriptome of an endangered plant, *T. chinensis*, under cold stress. Transcriptomic analyses revealed that 2,025 gene isoforms expressed differently at *p* < 0.05 and had a fold-change of at least 4; 1,437 of these genes were up-regulated and 588 were down-regulated by cold stress. Gene annotation also pointed to the signal transduction and transcription control of cold response genes of *T. chinensis* involving ROS.

Cold treatment induced the accumulation of stress-responsive messengers such as the ROS and subsequently activated the TFs signaling pathway for transcription regulation. An important signaling path for regulation of cold responsive genes in *T. chinensis* was MAPK signaling pathway and the most abundant TF families involved in cold response were NAC, WRKY, ERF, MYB, and bZIP. ROS were supposed to be accumulated and acted as a key messenger during the signaling process, whereas, excessive ROS may have also induced damage to cells. Enzyme activity analyses suggested that the POD, SOD, and CAT are enhanced to remove excessive ROS in leaf tissues of *T. chinensis*. Annotation of up-regulated transcripts further confirmed the response of the ROS scavenging system in *T. chinensis* to cold stress. Genes such as peroxidase and PHGPx were up-regulated in response to cold stress, and other anti-oxidative defense systems, such as starch and sucrose metabolism and thiamine metabolism were enhanced in response to cold stress.

## Author contributions

YL, XL, XY, HY, DL, and HL conceived and designed the experiments. XY, XH, and LM performed the experiments. DM and LM analyzed the data. YL, HY, XL, and HL contributed reagents and materials. DM and JH wrote and revised the paper. All authors read and approved the final manuscript.

### Conflict of interest statement

The authors declare that the research was conducted in the absence of any commercial or financial relationships that could be construed as a potential conflict of interest.
