# Supplementary material for: Transcriptomic Response of Chinese Yew (Taxus chinensis) to Cold Stress
Source: Front Plant Sci. 2017 Apr 28;8:468. doi: 10.3389/fpls.2017.00468 (PMC5408010; doi:10.3389/fpls.2017.00468)
Supplement: Supplementary file 2 [file DataSheet1.DOCX]

Supplementary figures of

**Transcriptomic response of Chinese yew (*Taxus chinensis*) to cold stress**

Delong MENG, XianghuaYU, Liyuan MA, Jin HU, Yili LIANG, Xueduan LIU, Huaqun YIN


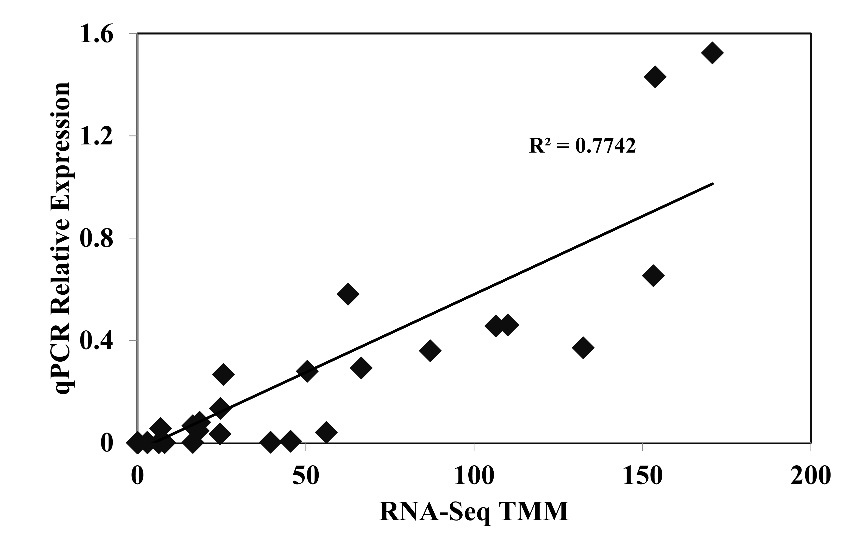


Figure S1 Correlation between qPCR determined gene expression and RNA-seq based TMM (Trimmed Mean of M-values) normalized counts number. Expression data obtained from different approaches correlated well with each other with Pearson’s correlation = 0.880, and *p* < 0.001).


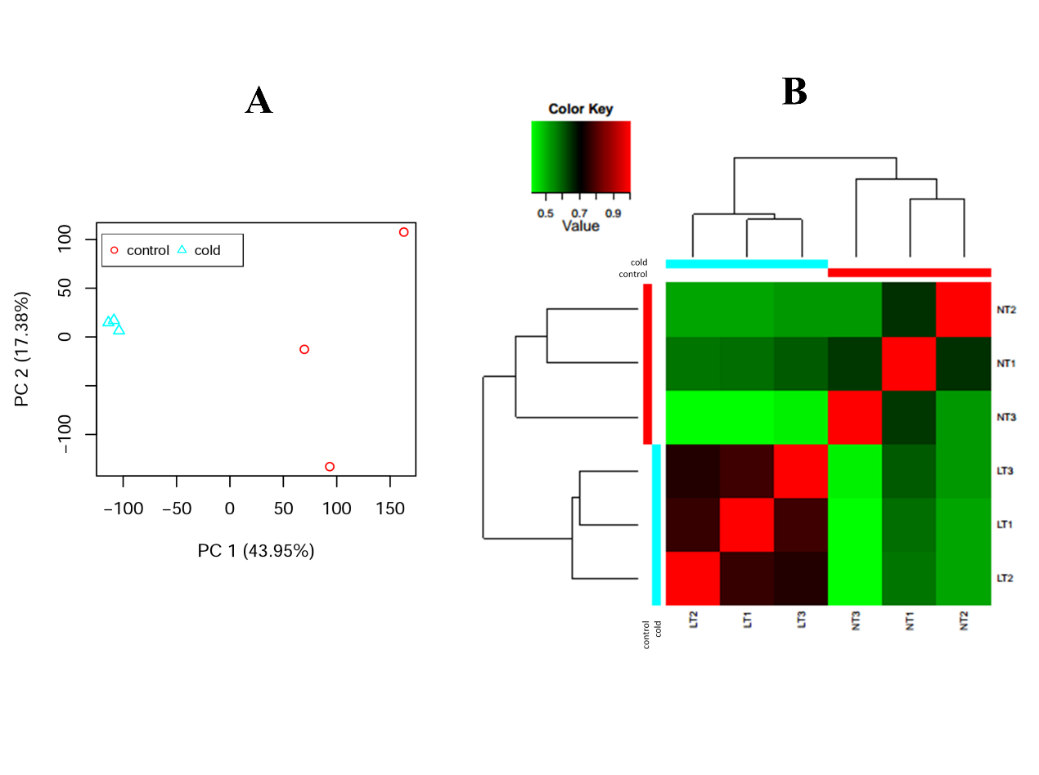


Figure S2, PCA (A) and correlation heatmap (B) of sample replicates. NT: samples collected from control plants, LT: samples collected from cold stressed plants.


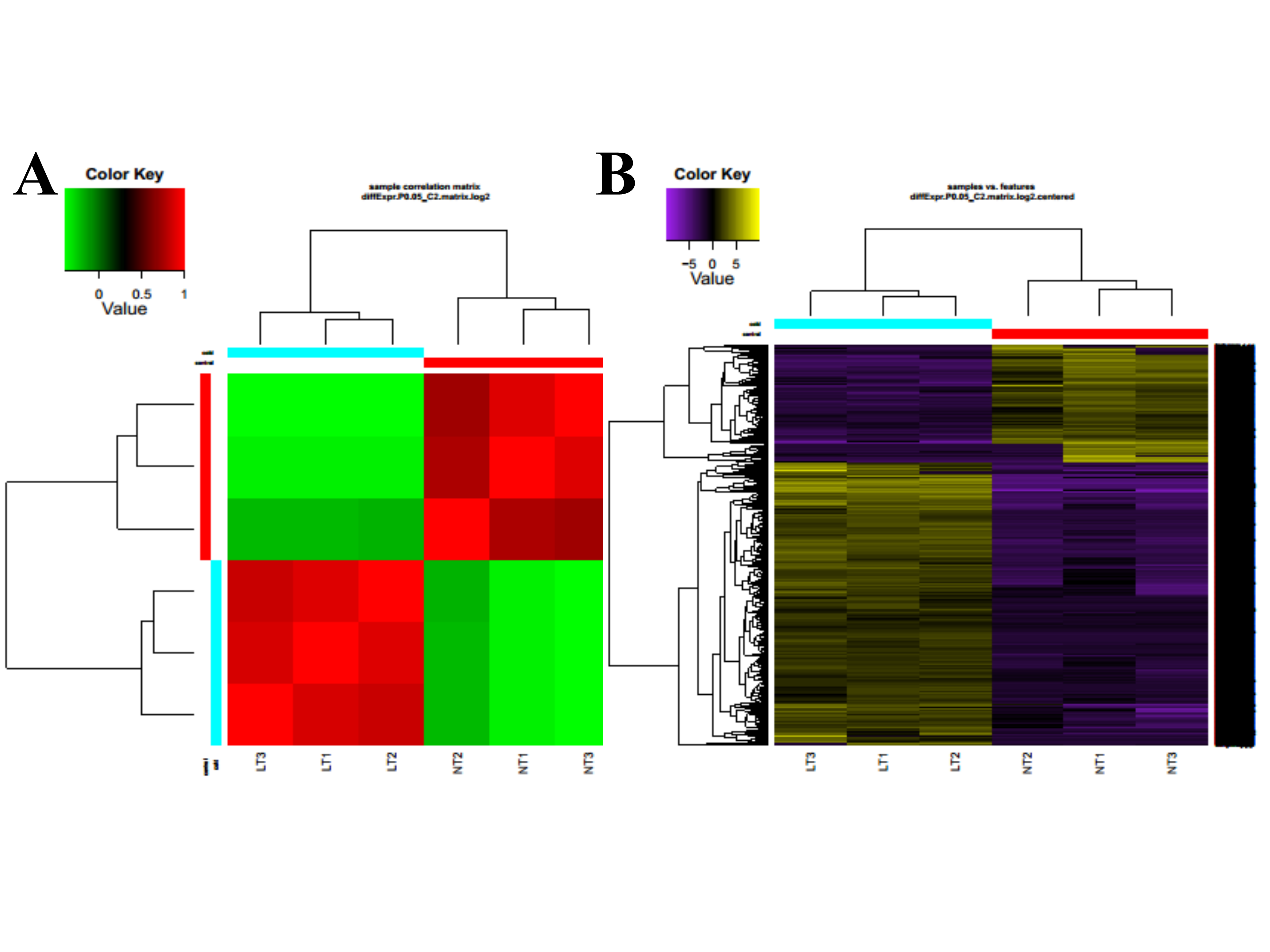


Figure S3, Sample correlation (A) and isoform correlation (B) heat map of differently expressed isoforms. Differently expressed meant isoforms that expressed differently between control and cold stressed samples at p < 0.05 level and had at least a folder change (FC) rate of 4 (|log_2_FC| > 2).


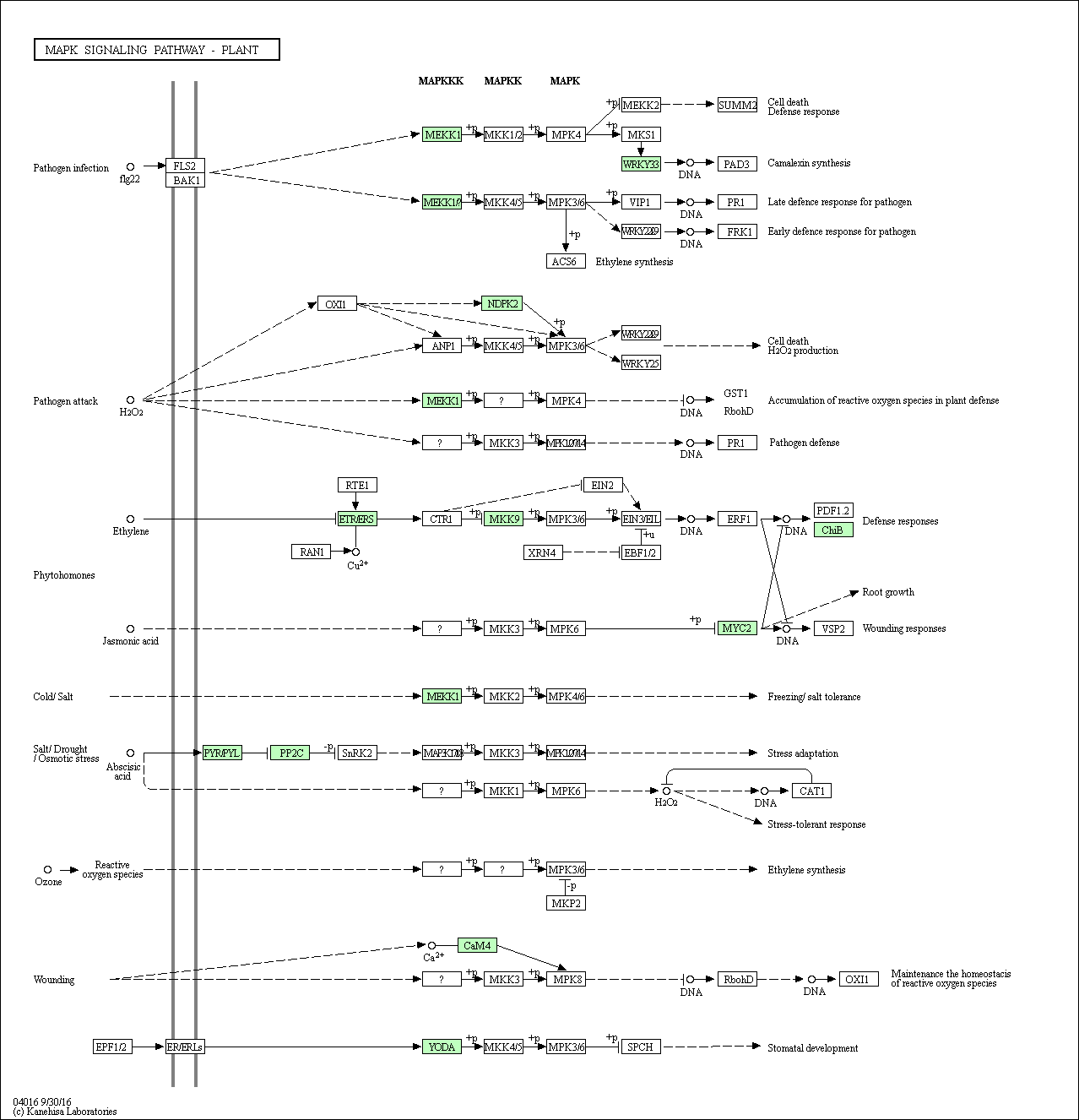


Figure S4 Plant mitogen-activated protein kinases (MAPK) signaling pathway (Ko04016) map. Green cells indicated the enzymes were up-regulated in leaf tissues of cold stressed *Taxus chinensis*.
